# Disposable and Low-Cost Colorimetric Sensors for Environmental Analysis

**DOI:** 10.3390/ijerph17228331

**Published:** 2020-11-11

**Authors:** Giancarla Alberti, Camilla Zanoni, Lisa Rita Magnaghi, Raffaela Biesuz

**Affiliations:** 1 Department of Chemistry, University of Pavia, Via Taramelli 12, 27100 Pavia, Italy; camilla.zanoni01@universitadipavia.it (C.Z.); lisarita.magnaghi01@universitadipavia.it (L.R.M.); rbiesuz@unipv.it (R.B.); 2 Unità di Ricerca di Pavia, INSTM, Via G. Giusti 9, 50121 Firenze, Italy

**Keywords:** colorimetric sensors, disposable devices, low-cost sensors, environmental analysis, in loco analysis

## Abstract

Environmental contamination affects human health and reduces the quality of life. Therefore, the monitoring of water and air quality is important, ensuring that all areas are acquiescent with the current legislation. Colorimetric sensors deliver quick, naked-eye detection, low-cost, and adequate determination of environmental analytes. In particular, disposable sensors are cheap and easy-to-use devices for single-shot measurements. Due to increasing requests for in situ analysis or resource-limited zones, disposable sensors’ development has increased. This review provides a brief insight into low-cost and disposable colorimetric sensors currently used for environmental analysis. The advantages and disadvantages of different colorimetric devices for environmental analysis are discussed.

## 1. Introduction

Current attention to environment systems derives from the evidence that people are disrupting the natural processes, and the degree of life quality, or life itself, is being threatened.

For example, the rapid population growth and the significant increase in energy consumption are signals of contingent problems. Emissions from automobiles, toxic particulate matter derived from combustion and incineration processes, waste production, or massive use of pesticides are the principal alarms.

Pollution is frequently associated with the diffusion of chemical toxics into the environment. Commonly, problems derive from an increase in the concentration of substances, which are naturally present in the environment due to their diffusion above the “natural” levels [1].

Many pollutants, such as organic compounds, drugs, dyes, pesticides, heavy metal ions, and anions can seriously affect air, water, and soils [2]. Moreover, small amounts of toxic substances can persuade ecological damage and irreversibly hurt people [3,4].

Attention in environmental monitoring continues to expand, thus the need for chemical analysis of environmental samples is growing. A significant percentage of the present monitoring is devoted to agreeing with legislation, and environmental analysis also provides scientific indication for further developments in the regulation matter [1].

Environmental monitoring provides interesting challenges to sensing technology: The development of quick, low-cost, sensitive, and selective methods for analyzing toxic pollutants is remarkable [5].

Usual classical instrumental methods are expensive and time-consuming; besides, restrictions in sampling and pretreatment standard techniques occur [6].

Classical and standard methods, especially for traces analysis, require some costly instruments, such as for toxic metal ions and inorganic contaminants: Atomic absorption spectroscopy (AAS), atomic fluorescence spectrometry (AFS) [7], inductively-coupled plasma-mass spectrometry (ICP-MS) [8], optical emission spectroscopy (OES) [9], X-ray fluorescence spectrometry [10]; or for organic pollutants: High-performance liquid chromatography (HPLC) [11], liquid chromatography-mass spectrometry (LC-MS), and gas chromatography-mass spectrometry (GC-MS) [12]. Trained personnel is also required to carry out operational procedures [13].

On the other hand, a necessity occurs for accurate, cheap, and on-site environmental pollutants monitoring. This goal can be achieved using sensing devices. Nevertheless, the development and use of emerging sensors are tentative due to technological and cultural obstacles [6].

In any case, with the progress of nanotechnology and materials science, several sensors and biosensors, based on nanomaterials such as nanoparticles (NPs) [14,15,16,17], quantum dots (QDs) [18], carbon nanotubes, and nanofibers (CNTs/CNFs) [19,20,21], nanowires [22], graphenes [23,24], etc., have been developed for environmental monitoring. For example, glassy carbon electrodes modified with nanomaterials are applied as electrochemical sensors [25,26,27,28,29]. Fluorescent NPs and QDs-based platforms allowed determinations, thanks to fluorescent quenching [30]. Metal-NP-based active materials are used for surface-enhanced Raman scattering analysis [31]. Unfortunately, this kind of sensor shows some limitations due to the time-consuming analysis, which is not useful for in situ determinations.

In contrast, colorimetric sensors seem promising for identifying pollutants in environmental samples thanks to their easy preparation, naked-eye sensing, fast detection, and pretty good sensitivity [32,33,34,35].

In this direction, the development of disposable and low-cost colorimetric sensors is key for a quick and in situ detection of environmental pollutants.

Disposable sensors are cheap and easy-to-use devices. They do not experience the memory effects, do not need pretreatment before their usage or cleaning between measurements. Due to the growing demand for in-situ analysis, the disposable sensors’ global market is rapidly increasing, particularly for medical diagnostics and environmental monitoring [13,36].

This paper comprehensively reviews disposable colorimetric sensors, recently described in the literature, providing a critical discussion about the advantages and disadvantages in their applications for practical, in situ, quick, and reproducible environmental analysis.

## 2. Detection and Image Analysis

Different kinds of detectors can be used for optical sensing, from the most current and low-cost devices (i.e., scanners or camera phones), to more specialized instruments, such as spectrophotometers and fluorimeters.

The office scanner is a shared device that provides fair resolution and guarantees the digitalized image; moreover, the image intensity is not affected by the external light. Scanners are widely employed since they can be portable (for example, pen scanners) and suitable for unskilled personnel.

Digital and cell phone cameras can also be used as detectors, with the advantage that they do not require any particular skills. One shortcoming is that digital and cell phone cameras cannot focus on too close objects (around 20 cm).

After digitalization, images can be analyzed using software or apps such as Adobe Photoshop, Microsoft Paint, DigitalColor Meter, Corel Photo-Paint, GIMPs. Digitalized images are then converted to color spaces: RGB, CMYK, grayscale, HSV, or CIE L*a*b* [37]. Depending on the color and hue of the image analyzed, the full information, or just one channel of the color space, can be used for data treatment.

The last group of detectors includes simple photometers [38] and homemade devices. For example, the portable color identifier described by Li et al. [39] or the transmittance colorimeter described by Ellerbee et al. [40]; both devices were assembled with components readily available in any electronic store.

In any case, because of the rapid expansion of the smartphone market, this approach for image collection could replace all currently available detection techniques. Besides, smartphones are user friendly, low-cost, and do not require highly technical know-how.

Some research groups developed apps to directly collect and process images with a smartphone without the need for computers; moreover, RGB apps for color space analysis are offered free of charge [41].

## 3. Chemometric Tools for Colorimetric Data Analysis

Colorimetric sensors produce large datasets with many variables, so for handling this wealth of data, various chemometric methods have been proposed. Mainly used are principal component analysis (PCA), hierarchical cluster analysis (HCA), and partial least square regression (PLS).

### 3.1. PCA

Principal component analysis (PCA) is an unsupervised chemometric method that simultaneously analyzes the entire multidimensional data set. It is a technique for reducing datasets’ dimensionality, increasing interpretability, and preserving as much statistical information while minimizing information loss. The PCA algorithm uses an orthogonal transformation to convert observable and related variables into a set of uncorrelated variables, called principal components (PCs). These, even if few components are sufficient to describe the dataset, and in this way, patterns and trends in the data can be easily visualized and described. PCA has been applied in many disciplines, and it has been recognized as a useful tool to interpret data generated by colorimetric sensors for environmental analysis [41,42].

**Figure 1:**
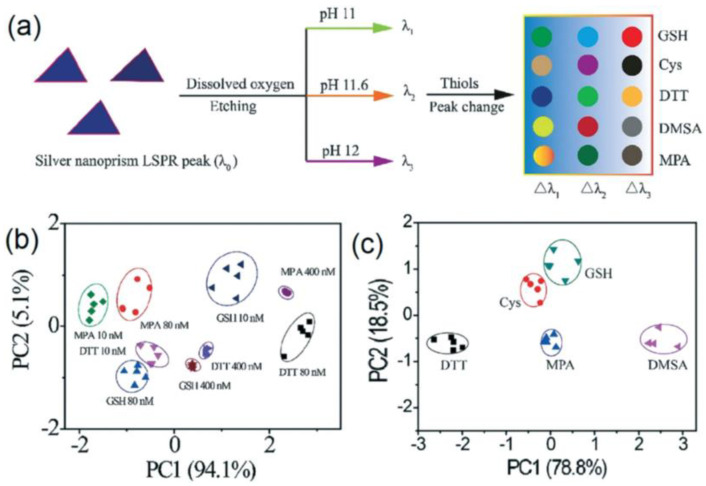
AgNPR-based colorimetric sensor for thiols: (**a**) sensing mechanism, (**b**) PCA (Principal Component Analysis) plot of the data matrix of three thiols at different concentrations, and (**c**) PCA plot of the data matrix of five thiols at a concentration of 400 nM [43].

As an example, Zhou et al. [43] developed a multichannel colorimetric sensor array based on the pH-induced etching of silver nanoprisms (AgNPRs) suitable for sensing five thiols (see Figure 1a): Cysteine (Cys), dimercaptosuccinic acid (DMSA), dithiothreitol (DTT), glutathione (GSH), and 3-mercaptopropionic acid (MPA), at trace concentrations (low to 10 nM). PCA is employed for data analysis. The PCA plots indicated the possibility to distinguish and identify three thiols in mixtures at different concentrations of 10, 80, and 400 nM (Figure 1b), and all the five thiols at a concentration of 400 nM in diluted fetal bovine serum (for simulating a real sample, Figure 1c). Other unknown samples are analyzed, and the five thiols are accurately identified, so the array seems promising for thiol sensing in biological and environmental matrixes.

More interesting PCA applications to colorimetric arrays with smartphone detection have been reported [44,45]. In both cases, the colorimetric analysis is used to quantify simultaneously different analytes, and the chemometric approach permitted a clear distinction between the analytes signals.

In summary, PCA has been recognized as an effective tool for data treatment of the results obtained by colorimetric sensor devices, as evidenced by the numerous papers on this subject [46,47,48,49,50,51,52,53].

### 3.2. HCA

Hierarchical cluster analysis (HCA) is a multivariate unsupervised analysis technique sometimes coupled to PCA for data treatment in colorimetric arrays [45,48,49,51,53,54]. Additionally known as hierarchical clustering, HCA aims to group subjects with similar characteristics into clusters. In HCA, two different strategies can be used: agglomerative and divisive. The first case is an iterative process; each data point is initially considered an individual cluster. At each iteration, the similar clusters merge with others until one single cluster is formed. This strategy is also called a “bottom-up” approach. In the divisive clustering (also defined top-down), a single cluster of all data is divided routinely into two smallest similar clusters; the process ends when only one cluster for each observation is obtained. The divisive algorithm is practically the inverse of the agglomerative one.

A measure of dissimilarity between sets of observations is needed for deciding which clusters can be combined (for agglomerative) or when a cluster should be split (for divisive). The general approach consists of using an appropriate metric (a measure of the distance between pairs of observations) and a linkage criterion that specifies the dissimilarity of sets as a function of the pairwise distances of observations.

The result of hierarchical clustering is often presented in a dendrogram (also called a “tree diagram”). The objects are organized in a row according to their similarities [55].

**Figure 2:**
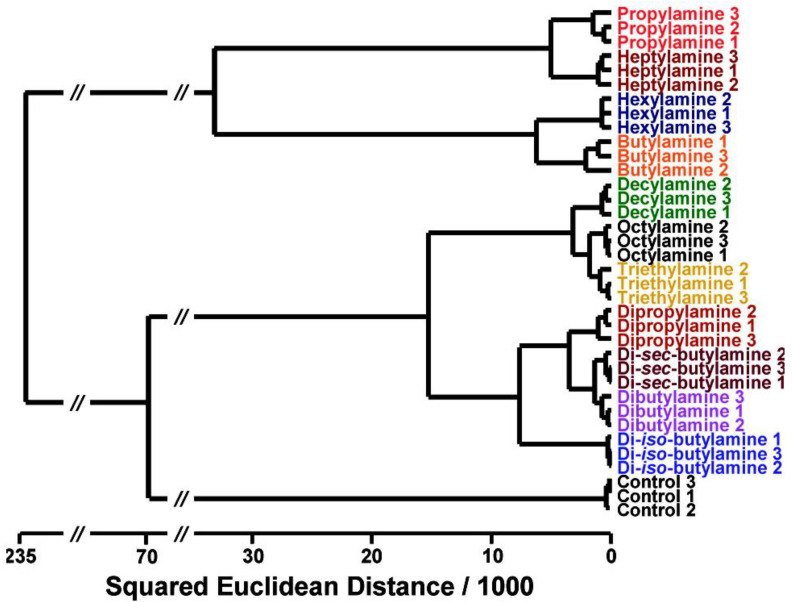
Dendrogram HCA (Hierarchical Cluster Analysis) for 11 structurally similar aliphatic amines using Ward’s method. No misclassifications were observed among the 36 trials. All experiments were run in triplicate [48].

As an example, Figure 2 shows the dendrogram reported by Bang et al. [48]. In this work, colorimetric sensor arrays are developed to identify and quantify toxic aliphatic amines. The HCA procedure permitted the complete identification of 11 structurally similar amines.

### 3.3. PLS

The multivariate regression PLS (partial least squares regression) is a supervised technique that combines characteristics of the principal component analysis (PCA) and multiple regression. The work of Gerlach and Kowalski [56] describes in detail the procedure of PLS. Thanks to its potential to extract information, PLS regression based on data obtained from colorimetric sensors is broadly used.

PLS is commonly applied to the simultaneous examination of two datasets, such as spectra or image parameters (i.e., RGB, HSV, etc.) and concentration. Based on different factors (latent variables), PLS creates a linear model y = X × b, allowing the prediction of the concentration (y) from spectra or image parameters (X); b includes the regression coefficients obtained in a calibration process.

In detail, X is the n × m matrix of measured responses obtained from spectra or images, n is the number of samples, and m is the number of wavelengths of the whole spectrum or all image parameters. y is the n × c concentration matrix of c analytes. b is the n × c vector of regression coefficients, which is obtained in the calibration. Knowing b, the calibrated model can predict new y concentrations from measured X spectra or image parameters of unknown samples.

Unlike unsupervised methods, like PCA and HCA, supervised techniques result in predictive models that permit qualitative (classification techniques) or quantitative analyses (regression methods). Among the latter, PLS is definitely the most famous regression tool for sensing applications.

This chemometric method is applied in a discrete number of works dealing with colorimetric sensors’ development in environmental analysis.

Below are some examples:

For the simultaneous analysis of multiple ions, Huang et al. [57] proposed a rapid and simple colorimetric method to detect metal ions and oxyanions based on gold nanoparticles functionalized with amino acids (amino acid-AuNPs) using PLS for data treatment. Taking advantage of the multivariate analysis by PLS, they can simultaneously quantify each ion in binary and ternary mixture of Cr(III), Cr(VI), and Fe(III).

Ferri et al. [58] developed a colorimetric array containing twelve dyes to detect and discriminate several organophosphorus pesticides in water. PLS model is applied for evaluating the influence of the concentration on the chromogenic array response. The PLS model shows a good agreement between the measured and predicted values, suggesting that the array could determine all the organophosphorus pesticides investigated.

Catechols are common substances used as pharmaceutical or intermediates in chemical synthesis. Despite the high value of catechols in these fields, the illegal discharge and accidental leak of catechols cause environmental pollution and harm human health. Wang et al. [59] proposed a colorimetric sensor array to identify and quantify 13 different catechols. The device is obtained using pH indicators and phenylboronic acid. In particular, PLS models are applied for the quantitative determination of each specific catechol’s concentrations.

In different works of Alberti and Biesuz [47,60,61,62], the PLS method is successfully applied to quantify different analytes simultaneously; in these studies, low-cost, disposable colorimetric sensors are used.

## 4. Materials for Disposable Colorimetric Sensors

Despite the development of research in material science, it is still impossible to produce a unique material meeting all the disposable colorimetric sensors’ requirements due to their wide range of applications.

This section reports an overview of some materials used as support for disposable colorimetric sensors. They can be grouped into four categories:

- Cellulose-based materials;
- Textiles and woven non-woven fabrics;
- Synthetic polymeric supports;
- Sol-gel materials.

The focus is on sustainability (recyclable, biodegradable, etc.), fields of application, cost, and other particular properties, reporting the different materials’ advantages and limitations.

### 4.1. Cellulose-Based Materials

Cellulose is a chain of glucose residues, and it is the main constituent of the plant cell wall. Cellulose is the most abundant widespread, and sustainable biopolymer used for numerous applications. Paper is a smart, cellulose-based material for disposable sensors thanks to its properties: It is low-cost, lightweight, flexible, and biodegradable. It can be easily functionalized by chemicals and is well-suited with low-cost techniques of sensors fabrication like printing. Paper also can be used to develop microfluidic devices [63,64,65].

The choice is based mainly on the device’s fabrication steps and the particular application area since the variety of paper available materials. In the development of colorimetric sensors and microfluidic devices, filter papers have been widely used in recent years to produce paper-based sensors.

Colorimetric paper-based sensors are among the most common, since this material gives a bright, high-contrast, colorless background for color change analyses [66].

For example, Mentele et al. [67] reported a paper-based device for three metal ions determination. Sensing is based on three dyes fixed on the paper: 1,10-phenanthroline for Fe(II), dimethylglyoxime for Ni(II), and bathocuproine for Cu(I). In particular, the wax printed device comprises of three zones: sampling, pretreatment, and detection. The pretreatment zone is the best feature of this device: masking reagents and reducing agents can complex interfering metals eventually present, and if necessary, change the pH. This device effectively detected the three cations with good sensitivity; moreover, it could be easily modified by introducing different dyes to determine other metal ions.

Carpenter et al. [68] proposed a sensor for hydrogen sulfide gas. The device consists of a moist paper impregnated with a Cu(II)/PAN (1-(2-pyridylazo)-2-naphthol) complex and NaOH. Water and NaOH help to adsorb and convert H_2_S gas to sulfide, increasing the sensitivity. Of interest is the sensor’s coupling with a handheld colorimeter faced to a smartphone, obtaining the direct H_2_S quantification, and only measuring the degree of color change. The sensor can be applied for a wide range of concentrations for industrial monitoring and oral hygiene, with LOD of about 30 µg/L, so it can be suitable to detect the average concentration of bad breath (80 µg/L) and the US OSHA permissible exposure limit (10 mg/L).

**Figure 3:**
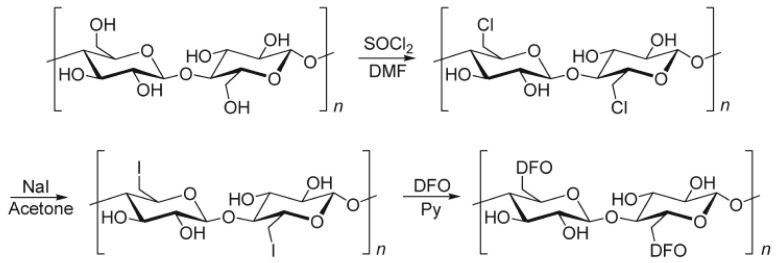
Preparation of papers functionalized by deferoxamine (DFO-papers) [43,69].

Alberti et al. [69] described a functionalized paper with deferoxamine (DFO) for sensing Fe(III) and V(V) in natural waters. DFO is selected thanks to the possibility of linking it on the paper and because its complexes with Fe(III) and V(V) are colored, thus a simple colorimetric sensor is prepared. The so-named DFO-papers are prepared following a method based on the procedure proposed by Takagai et al. [70,71]. It consists of a halogenation step, followed by introducing the DFO molecules (see Figure 3).

The colorimetric response of the sensory DFO–paper to the presence of Fe(III) or V(V) in solutions allowed the detection of these cations with the naked eye and their quantification. In particular, images acquired by a desktop scanner of the DFO–papers after exposure to aqueous solutions of Fe(III) or V(V) are used to quantify both metal ions. RGB parameters obtained by the scanner are processed using freeware software, followed by a simple chemometric procedure. This approach proved to be successful in determining Fe(III) and V(V) with a new ready-to-use and economical sensor.

Xu et al. [72] described devices obtained incorporating spirolactam rhodamine derivatives onto cellulose filter papers’ surface. They use these sensors to recognize Hg(II) ions in aqueous samples. The device showed an improved fluorescent emission intensity and a marked color change from colorless to pink after the complex formation with Hg(II) ions. The detection limit obtained is about 5 × 10^−5^ M; furthermore, this sensor displays high selectivity for mercury(II) over numerous environmentally critical metal ions.

Chen et al. [46] present a paper-based device for colorimetric sensing of dissolved NH_3_ and CO_2_. They functionalized a paper platform with CO_2_ and NH_3_ sensitive dyes showing the noteworthy performance in dissolved gases’ determination. The sensitivity of the device is limited by the RGB read-out from the scanned images. The responses of CO_2_ and NH_3_ are distinguished by applying the chemometric tool of Principle Component Analysis (PCA) on the RGB data. It is crucial to highlight that the results are obtained starting from solutions containing one analyte at a time. For real-world applications to actual samples, it will be nice to test the device with both gases’ mixtures at different concentrations.

Sicard et al. [73] proposed a tool for water quality monitoring. The sensor is a paper-based device (µPAD) that produced a colorimetric signal dependent on a specific analyte’s concentration (for example, the dynamic range for paraoxon and malathion is from 10^−8^ to 10^−6^ M). A cellphone equipped with a camera captured images of two µPADs: one put in contact with a water sample and the other, used as a control, with distilled water. The method has yet to be perfected, but it is promising and has a very high potential for improving water quality monitoring.

In addition to papers, there is a wide choice of other cellulose-based materials.

For example, cellophane can be used as a substrate for the fabrication of disposable and low-cost devices. Cellophane is a thin, transparent, renewed cellulose film obtained from sodium cellulose xanthate. It is transparent to visible and UV-light; it is not porous but encloses several capillaries filled with solutions during its swelling [74].

**Figure 4:**
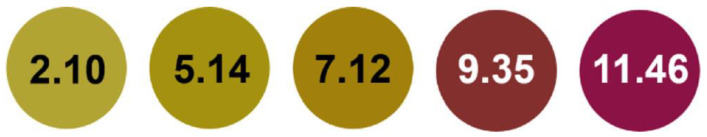
Colors of the curcumin-cellophane sensors after exposure to standard pH solutions (Radelkis Kft., Budapest, Hungary; pH 2.10, 5.14, 7.12, 9.35, and 11.46 (±0.03 at 25 °C)) [75].

Pávai et al. [75] developed a curcumin-colored cellophane test strip for pH measurements. Strips are simply obtained by immersion of 2 × 2 cm pieces of cellophane in aqueous solutions of E100-curcumin, (1E,6E)-1,7-bis(4-hydroxy-3-methoxyphenyl)-1,6-heptadiene-3,5-dione) for 24 h, then washed with ultrapure water and dried at room temperature. The color of the sensor change from yellow at low pH to brown-red to the high values, as reported in Figure 4.

Based on readily available materials, this sensor can provide an effective strategy for in situ pH determination.

The need for biodegradable films for packaging, sorbents, and sensors has encouraged the production of novel materials derived from natural sources, mostly agricultural byproducts. Sugarcane bagasse is cellulose-rich waste from the sugar industry, so it can be an excellent candidate for obtaining cellulose-based devices. Guo W. et al. [76] presented a novel cellulose-based colorimetric device for selective sensing of Ag^+^ and Cu^2+^. It is suitably obtained by grafting the 2,5-dithiourea (DTu) onto bagasse-pulp cellulose via a one-pot reaction. After exposure to metal-ion aqueous solutions, the cellulose-DTu device exhibits a white to yellow-red and white to light grey color change in the presence of Ag^+^ and Cu^2+^, respectively. Other cations, such as Al^3+^, Ba^2+^, Ca^2+^, Cd^2+^, Hg^2+^, K^+^, Li^+^, Mg^2+^, Mn^2+^, Na^+^, Ni^2+^, Pb^2+^, Zn^2+^, do not interfere in the measurements since they do not produce any color change.

**Figure 5:**
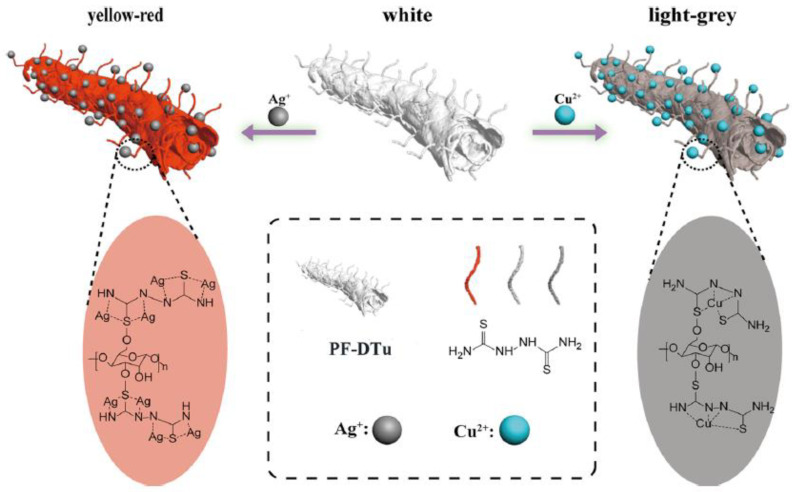
A possible mechanism for the complexation reaction of Ag+ and Cu^2+^ with cellulose-DTu (2,5-dithiourea) [76].

Figure 5 shows a possible mechanism of the complexation reactions between Ag^+^ or Cu^2+^ and the cellulose-DTu.

This sensor features the advantages of rapidity, easiness, and remarkable selectivity for Ag^+^ and Cu^2+^ sensing. An evident color change from white to yellow-red (for Ag^+^) and light grey (for Cu^2+^) in just 5 s is demonstrated.

Nanocellulose made from nanofibers, nanocrystalline, or bacterial can be used in disposable sensors as a biodegradable substrate. Cellulose nanofibers (CNFs) are isolated and purified using different chemical or mechanical processes, starting from cellulose fibers of lignocellulosic resources, such as wood and agricultural residues [77].

**Figure 6:**
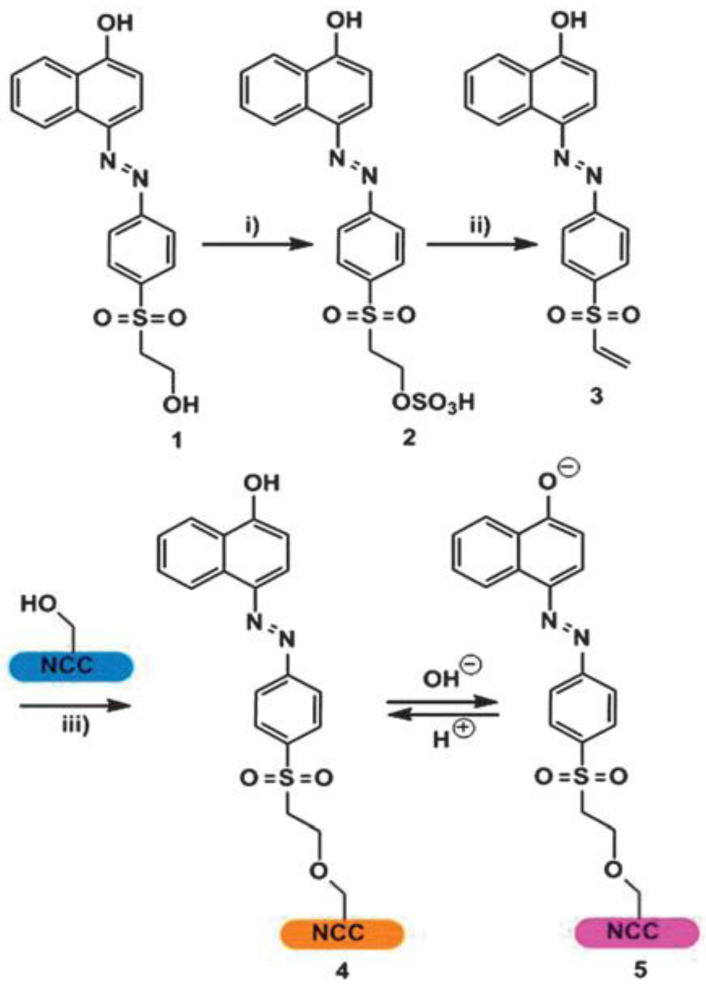
Synthesis of the nanocellulose dye film: (i) 96% H_2_SO_4_, 45 min, 45 °C, yield: 7.4%; (ii) NaOH 1 M until pH = 10; (iii) nanocellulose suspension in water, yield: 37% [78].

Chauhan et al. [78] described a one-step synthesis of a nanocellulose functionalized with a dye for optical pH sensing. The nanocellulose is obtained by hydrolysis (with 64% sulfuric acid for 45 min at 45 °C) of commercial microcrystalline cellulose from cotton fibers. The dye employed belongs to the class of Remazol dyes [79]; it is covalently attached to the nanocellulose material according to the reaction synthesis shown in Figure 6.

The thus obtained nanocellulose–dye product gave stable suspensions that change color from orange to purple, increasing the pH from acid to alkaline. A piece of the nanocellulose–dye film is taped on a plastic strip, thus obtaining a disposable stick that responds reversibly and rapidly to pH changes.

### 4.2. Textiles and Woven Non-Woven Fabrics

Textiles represent an attractive class of substrates for fabricating smart chemical sensors. Chromic textiles are fabrics or fibers that change color depending on the type of external stimulus. Several works have been addressed to develop these textiles since their use as smart materials [80]. Of particular interest for environmental monitoring could be ionochromic textiles, but, until now, very few studies are reported for application in this field.

**Figure 7:**
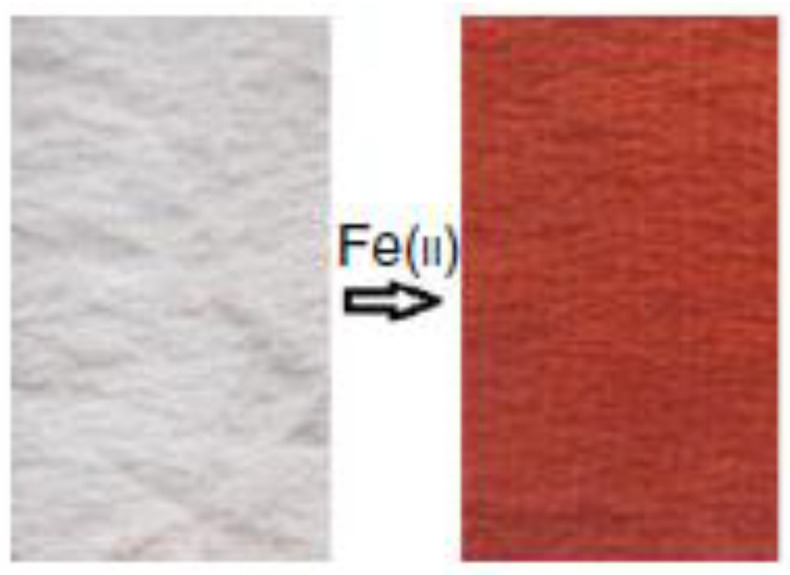
Fe(II)-sensitive color changes of silk fabric dyed with 1,10—phenanthroline (PHE) [81].

A noteworthy paper [81] presents an ionochromic textile for the detection of Fe(II). This material is produced by dyeing 1,10—phenanthroline (PHE) on silk. The interaction with Fe(II) produces an evident color change from white to red, as shown in Figure 7. The best performances were obtained after contacted with 8 mg/L of Fe(II) solution at neutral pH for about 15 min. This ionochromic textile may find broad applications in different fields, such as Fe(II) detection, magic toys, anti-counterfeiting materials, and bionic silk flowers.

Hydrogen peroxide (H_2_O_2_) plays an essential role in chemistry, biology, environmental, food, and industrial analysis, thus detecting H_2_O_2_ concentration with high selectivity and sensitivity is crucial.

An interesting study focuses on fabricating a flexible, textile-based, disposable biosensing device fabricated via a photolithography technique. It is applied to develop a biosensor for the colorimetric detection of H_2_O_2_ [82]. The analysis is based on the reaction of the dye 2,2′-Azino-bis(3-ethylbenzthiazoline-6-sulfonic acid) diammonium salt (ABTS), that, after oxidation by Horseradish peroxidase (POX) in the presence of H_2_O_2_, produced a purple colored product. After contact with H_2_O_2_, photographs of the colored material are taken and the color intensity analysis is done using the Image J program for H_2_O_2_ quantification. The system could work until 50 °C temperatures without any loss in the signal and in a pH range between 3 to 7.

**Figure 8:**
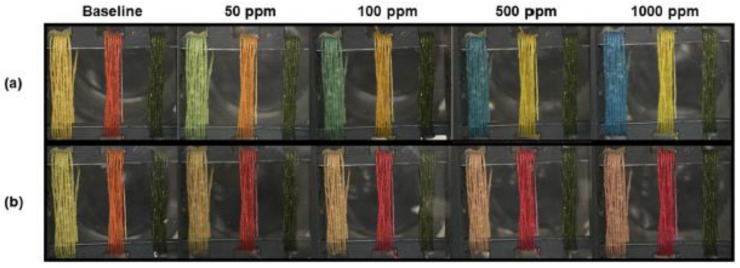
Optical images of BTB (bromothymol blue), MR (methyl red), and MnTPP (5,10,15,20-Tetraphenyl-21H,23H-porphine manganese(III) chloride) devices for different concentrations of (**a**) NH_3_ or (**b**) HCl [83].

The recognition of volatile gases in the environment is crucial for monitoring food spoilage or allergens, human health, and guarantee public and workplace safety. A method for obtaining a stable entrapment of dyes on a thread substrate has been proposed for gas sensing [83]. The dyes used in this work are 5,10,15,20-Tetraphenyl-21H,23H-porphine manganese(III) chloride (MnTPP), methyl red (MR), and bromothymol blue (BTB). A smartphone is employed to extract the red (R), green (G), and blue (B) parameters from the acquired images of the thread before and after contact with the gaseous analyte. This sensor can determine from 50 to 1000 ppm of ammonia and hydrogen chloride vapors (i.e., components commonly found in cleaning supplies, fertilizer, and materials production). Examples of gas sensing are shown in Figure 8, where optical images of BTB, MR, and MnTPP devices for different ammonia or HCl concentrations are reported.

The use of these devices as washable gas sensors integrated into textiles represents a further exciting application, and they belong to the new generation of smart textiles for environmental monitoring of various volatile gases.

**Figure 9:**
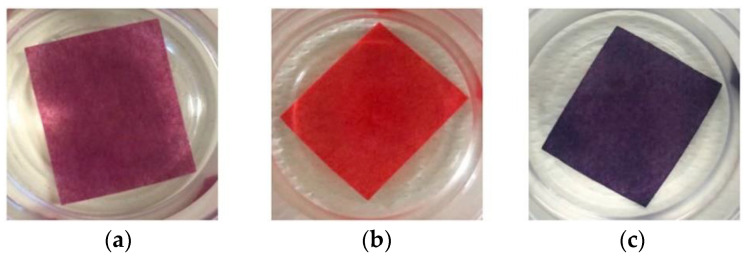
Colour change of Aliz-CC@ before (**a**), and after equilibration in Al(III) solution (**b**) or Fe(III) solution (**c**) [60].

The Colour Catcher^®^ (CC, Henkel Italia, Milano, Italy) and similar products have been in the washing powder market for several years. Their success is because their use in the washing machine can prevent color run problems. Each Colour Catcher^®^ packet contains 12 to 20 sheets of 11 × 25 cm, made of a material that looks relatively rigid, but, once wetted, it becomes soft, like a textile. The CC sheet express sequestration capability towards ions and molecules once released by clothes, even in the presence of surfactants and fabric softeners. Tissue dyes are often anionic, so, in some recent papers [60,61,62], the CC sheet is tested as support for preparing colorimetric sensors. The study reported in [60] describes an inexpensive and disposable device for Fe(III) and Al(III) sensing, prepared by dyeing an aqueous solution of Alizarine Red S on 2 × 2.5 cm pieces of CC for 3 h. The obtained sensor is called Aliz-CC@, and, when kept in water solutions, it is stable for at least three days. Fe(III) and Al(III) interactions with the Alizarin Red S-based sensor produce a change in the absorption spectra of the anchored dye, enabling to observe a naked-eye color variation (see Figure 9). The Aliz-CC@ device can sorb both cations in about 1–2 h. By this sensor, it is possible the simultaneous determination of Fe(III) and Al(III) applying the chemometric multivariate regression PLS (partial least squares regression) to the UV–vis spectra of the Aliz-CC@, registered after equilibration of the sensor in mixtures of Fe(III) and Al(III) at pH = 4.5. The models obtained demonstrated their ability to predict the concentration of both cations in aqueous samples, so the sensor is promising for the simultaneous determination of Fe(III) and Al(III) in environmental and biological samples.

The same CC sheet is also used to develop other colorimetric devices for different metal ions and sulfur and thiols detection. In particular, Eriochrome Black T (EBT) and 1-(2-pyridylazo)-2-naphthol (PAN) were employed, the first for Ca(II) and Mg(II), and the last for Co(II), Ni(II), and Zn(II) sensing [61]. These devices, respectively named EBT-CC and PAN, are simply obtained, placing portions of CC in aqueous solutions of the dyes. The best experimental conditions for each sensor’s preparation are found. Similarly to the previously described Aliz-CC@, PLS (partial least squares regression) is applied to correlate the device’s UV–vis spectra with the metal ion concentration. The PLS models obtained proved their ability to predict the investigated metal ions’ concentrations in synthetic and actual aqueous samples.

**Figure 10:**
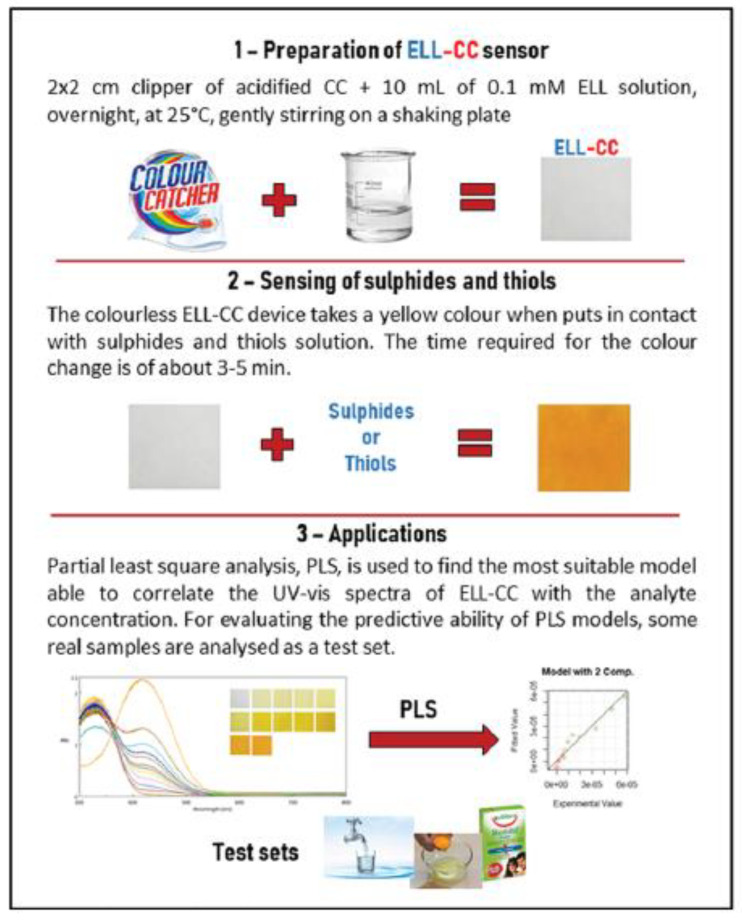
Preparation and application of the ELL-CC sensor [62].

Due to the biological and environmental relevance of thiols and sulfides, the scientific community drew the attention of their determination. In this field, a disposable and low-cost sensor is proposed [62]. With the same procedure reported above, the 5,50-dithiobis(2-nitrobenzoic acid) (Ellman’s reagent, ELL), a specific reagent for sulfides and thiols, is fixed on pieces of CC. The device obtained is named ELL-CC. Figure 10 shows a schematic of the work. As shown in the picture of Figure 10, the device assumes a yellow color after equilibration with solutions of sulfides and thiols. This property has been exploited to develop an optical sensor since the color intensity can be correlated with the analyte sorbed concentration. The PLS models obtained proved their predictive capabilities, so the device is promising to a fast and economical determination of sulfurs and thiols in actual environmental and biological samples.

### 4.3. Synthetic Polymeric Supports

Synthetic polymers are usually cheap and enable fast mass production and possibly prototyping. Due to the wide assortment of polymers with different properties, such as flexibility, transparency, stretchability, etc., they can be adequate supports for disposable sensors.

For example, thermoplastics are thermosoftening, thus they can be molded and reformed over a specific temperature (glass transition). Classical thermoplastics for disposable sensors are poly(methyl methacrylate) (PMMA), polypropylene (PP), polystyrene (PS), poly(ethylene terephthalate (PET), and poly(tetrafluoroethylene) (PTFE). Unlike other polymers, they offer chemical resistance to organic solvents, lower gas impermeability, a wide range of stiffness, and reduced biofouling. Thanks to these properties, thermoplastics are efficient substrates for disposable devices [36]. The following are some examples.

Halogenated organic compounds (HOCs) such as trihalomethanes (THMs) are known to cause adverse health effects, so novel methods can rapidly monitor the potential THMs concentration of source and finished drinking water, and could help mitigate potential health worries and improve the quality of waters. Wujcik et al. [84] proposed a colorimetric sensor based on nanoporous polypropylene fiber membranes for trihalomethanes determination at environmentally relevant levels (ppb-scale). In particular, a syndiotactic polypropylene (sPP) nanoporous electrospun fiber membrane is prepared and used as a sorbent for THMs in the headspace of a heated water sample. The colorimetric Fujiwara reaction [85] is applied as a detection reaction. The membrane is then tested for preconcentration and assay of THMs from contaminated water samples using image intensity analysis. Thanks to the positive results, this device holds potential as a portable tool for economic, on-site environmental analysis.

The work of Salcedo and Sevilla [86] presents a Hg(0) sensor based on cuprous iodide (CuI)/polystyrene composite immobilized on a cellulosic substrate: This device showed a reddish color in the presence of mercury vapors. Images of the sensor are acquired by a smartphone camera and analyzed in RGB color space by ImageJ (open-source image processing program). The colorimetric measurement is based on the formation of a red complex between Hg and CuI, according to the following reaction: 4 CuI + Hg ⇆ Cu_2_[HgI_4_] + Cu.

The method is validated by recovery tests, spiking an air sample with gas mixtures standard at different Hg concentrations: the recovery is satisfactory, ranging between 95 and 112%. The device is suitable for measuring gaseous mercury at ppb levels.

Besides thermoplastics, other synthetic materials were developed to prepare colorimetric devices.

For example, Deng et al. [87] reported a fibrous material obtained incorporating the dye 4-(2-pyridylazo)-resorcinol (PAR) onto polyacrylonitrile (PAN) fiber for the detection of some heavy metal ions, such as Cu(II), Hg(II), Ni(II), and Pb(II). The fiber is prepared using an MW-assisted method, and it is named PAN_MW_-PAR.

**Figure 11:**
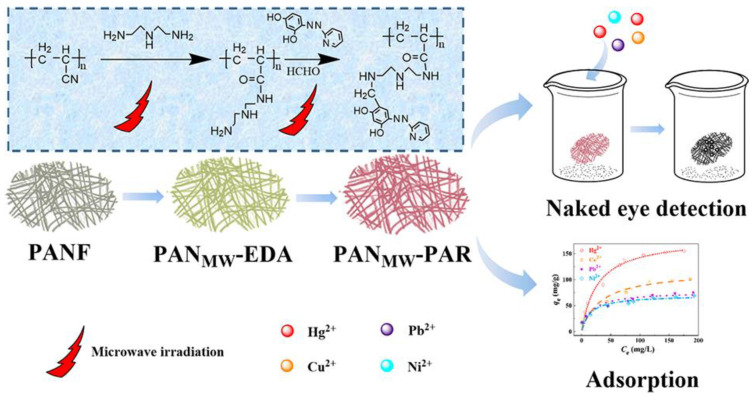
Scheme of PAN_MW_-PAR synthesis and sorption isotherms for Cu(II), Hg(II), Ni(II), and Pb(II). [87].

Figure 11 shows a scheme of fiber preparation and the sorption isotherms for the selected cations. Results demonstrated that PAN_MW_-PAR fibers after contact with the metal ions solution show a color change from red to black, and the sensitivity is pretty high. The sorption capacity for the investigated metal ions, especially for Hg(II), is excellent. PAN_MW_-PAR fibers are easily regenerated with EDTA solution and can be reused after 10 sorption cycles. No response is observed in solutions of other abundant cations, such as Ca(II), Mg(II), and Al(III), showing the remarkable selectivity of the fiber for heavy metal ions.

**Figure 12:**
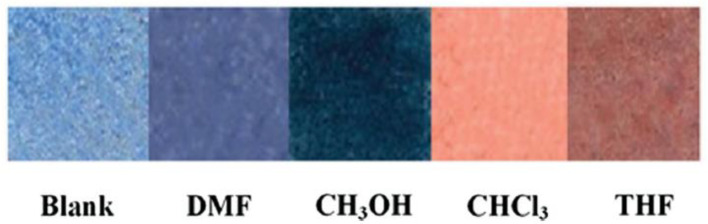
Photographs of the PDA/graphene films after exposure to dimethylformamide (DMF), methanol (CH_3_OH), chloroform (CHCl_3_), and tetrahydrofuran (THF) vapors for 2 min [89].

Exposure to VOCs (volatile organic compounds) can severely affect human health, so VOCs’ detection and identification are essential. The application of colorimetric techniques is a new strategy to overcome many limits of traditional gas sensing methods [88]. A chemosensor, obtained by a self-assembled polydiacetylene (PDA)/graphene stacked composite film, is developed to quantify VOCs [89]. Graphene is used as support for the PDA-based sensor since ordered monolayers of PDA are efficiently formed on the large surface of a graphene sheet; moreover, graphene is highly transparent in the UV–vis range, thus the signal of the visual chromatic change can be efficiently identified by spectroscopy. For testing the ability to detect VOCs, the PDA/graphene paper is exposed for 2 min to organic vapors in concentrations ranging from 0.01% to 20% (percent by volume). Figure 12 shows PDA/graphene film images after contact with the VOCs, demonstrating an evident color change detectable just by the naked eye, so without other instruments.

Formaldehyde is widely applied as a base chemical to manufacture building materials and household products. Nevertheless, it is a dangerous air pollutant, and extended exposure to this contaminant can cause serious health problems. A new and simple strategy for colorimetric sensing of formaldehyde (HCHO) using a nanofiber-based functional strip is presented [90]. In particular, the polyethyleneimine (PEI) functionalized poly(methyl methacrylate) nanofiber membrane is fabricated by electrospinning method. The functionalized nanofiber showed a color change from white to yellow after interaction with formaldehyde. It is observed that this sensor could detect down to 75 ppm of formaldehyde at room temperature. On evaluating possible interferents, such as ethanol, DMF, DCM, acetone, acetic acid, and chloroform, it is shown that no color variation is verified, confirming the sensor’s selectivity for formaldehyde. The mechanism involves the nucleophilic addition reaction between formaldehyde and the primary amine of PEI. The nanofiber format favored the reaction due to the high surface area. This sensor provides low-cost sensing of formaldehyde at low concentrations, and it is advantageous for environmental monitoring and food quality assessments.

Among the most popular polymers in colorimetric sensors, acetylcellulose and polyvinyl chloride (PVC) are widely applied. The transparent triacetylcellulose is a completely acetylated cellulose polymer. It can be employed as a raw material to prepare plastics, fibers, and films such as photo films. Moreover, the triacetylcellulose film can be used as porous support for colorimetric sensors. Tavallali’s and Safavi groups of two Iranian Universities developed several triacetylcellulose-based disposable optical sensors for metal ions, using, as starter material, waste photographic film tapes, immobilizing on the membrane different dyes [69,70,71,72,73,91,92,93,94,95]. All these studies adopted the same procedure to prepare the sensors. The photographic films were previously treated with sodium hypochlorite to remove the colored layer. The obtained transparent films were contacted with a suitable dye solution in ethylene diamine for a few minutes at room temperature, then washed with double-distilled water for removing excess ethylene diamine and the loosely trapped dye. The prepared membranes were kept in water until their use.

The goal of these kinds of devices is to use waste material with excellent optical and mechanical properties as support for dyes immobilization. These membranes respond to metal ions by changing color, and the signal can be revealed by nacked-eyes or spectrophotometrically.

By adapting the above-reported procedure, a colorimetric sensor for Cu(II), Cd(II), Zn(II), and Hg(II) sensing has been prepared by immobilization of dithizone (DTZ) on a triacetylcellulose film (mem-DTZ) [47,74]. The membrane responds to the divalent cations by changing color reversibly. The metal ions sorption on the mem-DTZ has been characterized by kinetics, isotherms, and profiles in the function of the pH. The paper highlights methods for assessing the cations’ concentrations, either individually or in a mixture, in unknown samples. After equilibration of the mem-DTZ with different cations solutions, UV–vis spectra, and RGB parameters of pictures obtained by a desktop scanner are acquired and correlated with the metal ions’ concentration in solutions. A single metal ion determination is performed by applying principal component analysis (PCA) to the pictures’ RGB parameters; otherwise, UV–vis spectra of a mixture of two cations are subjected to partial least squares (PLS) regression. The sensor’s applicability to real samples has been proved by analyzing the four metal cations in a certificate material (Sewage Sludge CC136A), drinking water, and white wine samples.

### 4.4. Sol-Gel Materials

The sol-gel method allows for the formation of ceramics and glassy materials at temperatures much lower than those used in traditional melting techniques. The first study about sol-gels appeared about 150 years ago, but the real development of this technology and its applications occurred in the last few years.

Sol-gel methods can produce different kinds of materials: monoliths, thin films, or fibers. The strict control of the experimental conditions during the production process yields materials with well-defined properties [96]. The sol-gel-based materials may provide excellent substrates for various devices such as colorimetric sensors; some examples are here summarized.

**Figure 13:**
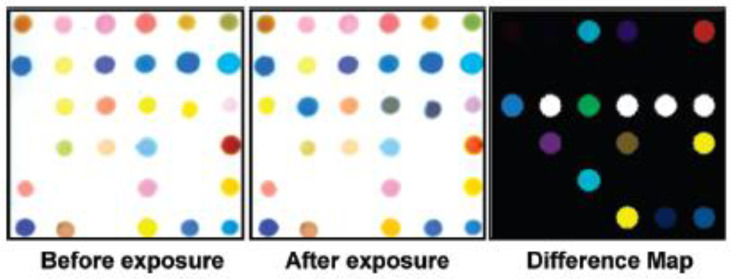
Images of the colorimetric array before exposure, after 2 min of exposure to ammonia at its IDLH (immediately dangerous to life or health) concentration (300 ppm) at 298 K and 50% relative humidity and the generated color difference map [54].

A low-cost, colorimetric array for sensing TICs (toxic industrial chemicals) has been developed [54]. The colorimetric sensor array consisted of 36 different dyes printed on a nonporous PET (polyethylene terephthalate) film. The indicator classes for this colorimetric sensor array include metal ion-containing dyes (e.g., metalloporphyrins), pH indicators, vapochromic/solvatochromic dyes, and redox-sensitive metal salts. The device is printed through an array of 36 floating slotted pins by dipping into the inkwell and transferring to the PET film. The sol-gel obtained is kept under a slow nitrogen stream for at least three days before any analysis. Color difference maps are obtained by subtracting the array’s digital image before exposure from that after exposure, so each spot is characterized by its RGB color values (see Figure 13). Chemometric analyses are carried out. In particular, hierarchical cluster analysis (HCA) using the minimum variance method (Ward’s method).

The sensor array can discriminate among 20 TICs at their permissible exposure limit (PEL) concentrations, with limits of detection in the few µg/L range (well below the PEL). Moreover, the array is not affected by various potential interfering agents, and it shows good stability and reproducibility.

Sensitive detection of trimethylamine (TMA) both in aqueous and gaseous phases is obtained using a heap colorimetric sensor array [97]. This device incorporates three different kinds of colorants: metal-containing dyes (i.e., Zn(II) metalloporphyrin), pH indicators, and dyes with large permanent dipoles (i.e., vapochromic dyes).

**Figure 14:**
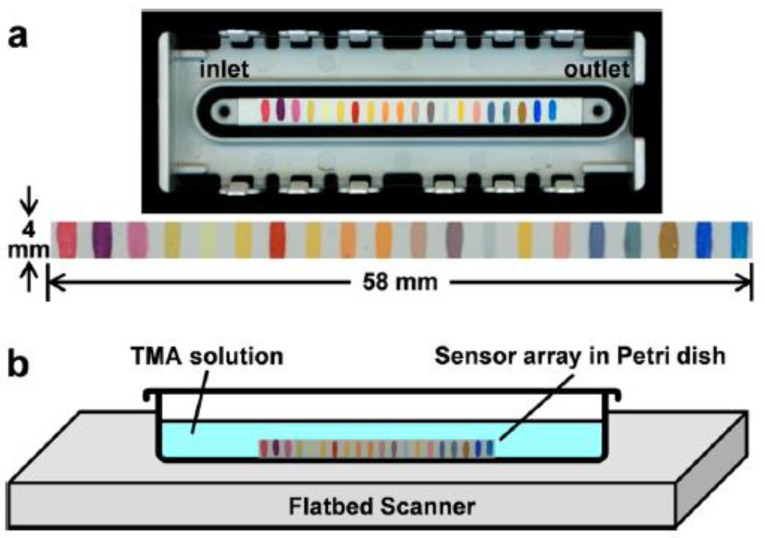
Colorimetric sensor array for trimethylamine (TMA) detection. (**a**) Linearized 20-element sensor array for vapor detection; (**b**) schematic of the experimental setup consisting of a closed Petri dish containing 10 mL of a buffered aqueous TMA solution, an array positioned in the solution, and an ordinary flatbed scanner for imaging [97].

Highly porous sol-gel formulations are used to better respond to gaseous analytes and the ideal hydrophobicity of the matrix, minimizing the colorants’ leaching during analysis. The 20-element arrays are linearized for improved gas flow path. It is printed and then attached to a polycarbonate cartridge, providing an ideal flow path for analytes and a flow volume of <180 μL (Figure 14).

As a response, digital pictures of the arrays are acquired before and after exposure to aqueous solutions or gas mixtures; from changes in RGB values of each spot, color difference maps are produced.

Characteristic color change patterns permitted easy discrimination among different trimethylamine concentrations. This array showed excellent reversibility with the possibility of distinguishing trimethylamine from similar amine odorants.

Chemical, biological, radiological, nuclear, and explosive agents pose significant threats in the twenty-first century, particularly for armed forces and first responders (i.e., police, firefighters, and other emergency personnel). Thus, quick, selective, and sensitive detection systems are required to identify and quantify specific compounds. In particular, there is a need for low-cost disposable sensors that are sensitive, stable, and reliable [41].

A couple of examples of sol-gel based sensors in this field are summarized in the following.

**Figure 15:**
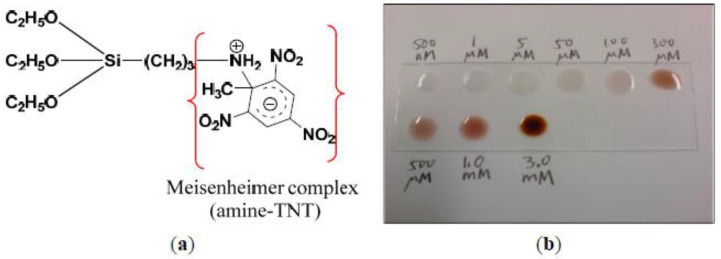
Functionalized Silica Nanoparticles for TNT detection. (**a**) The complex between the amine group and TNT molecule and (**b**) a gradual change in color as the TNT concentration increases [98].

A sensing device for selective detection of 2,4,6-trinitrotoluene (TNT) is obtained by functionalization of silica nanoparticles (NPs) with 3-aminopropyl-triethoxysilane (APTES) [98]. The amine anchored to the silica nanoparticles’ surface (SiO_2_-NH_2_) behaves like a probe for TNT molecules. A colorimetric change from the green of SiO_2_-NH_2_ nanoparticles towards red is observed after the reaction with TNT. As the TNT concentration increases, a gradual change to a dark red color can be observed, as shown in Figure 15. The naked eye’s detection range is limited to 100 μM, but using optical instruments makes it possible to detect lower quantities (about 10^−12^ M).

Thanks to the pretty good results, this device has the potential to further progress for easy-to-use and low-cost sensing applications.

A colorimetric sensor is developed for the sensitive gas detection of cyclohexanone, a volatile marker of some explosives, such as RDX (1,3,5-trinitro-1,3,5-triazinane) and HMX (1,3,5,7-tetranitro-1,3,5,7-tetrazocane) [99]. It consists of silica-dye composite microspheres prepared using ultrasonic and aerosol-gel synthesis, comprising the hydrolysis of ultrasonically sprayed organosiloxanes at 150 °C, with the adding of the ketone-responsive indicator dye, pararosaniline. With this procedure, nanoporous microspheres with high surface area (~300 m^2^/g) are prepared and used as colorimetric inks. Indeed, the sensor inks are dropped on cellulose paper, and the so obtained colorimetric strip allowed for a quick quantification of gaseous cyclohexanone in about 2 min, with a LOD around 150 ppb. The sensor displayed high specificity towards cyclohexanone against humidity and other common solvents classes, such as acetonitrile, ammonia, ethanol, ether, and ethyl acetate. This kind of device could be an alternative for sensing explosives.

A similar approach is applied to develop a colorimetric sensor array to identify aliphatic amines, pollutants often present in industrial wastewater effluents, and agricultural drainage because of their extensive use in several applications [48].

Sixteen different nanoporous pigment microspheres are prepared by an ultrasonic-spray aerosol-gel synthesis, starting from dyes and silica.

**Figure 16:**
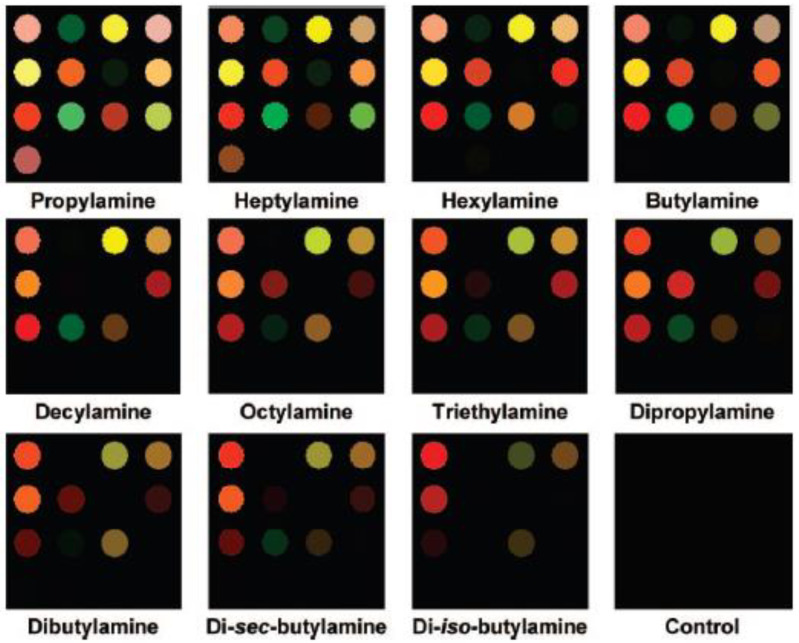
Color profiles of 11 similar aliphatic amines after equilibration with the aerosol-gel-based colorimetric sensor arrays [48].

The colorimetric sensor arrays are obtained by printing the prepared inks onto standard chromatography paper. The arrays are successfully tested for the identification and quantification of 11 toxic aliphatic amines. As evident from the images registered by a scanner reported in Figure 16, the sensor arrays showed a different color change for each amine. Subtracting the image obtained before and after exposure to amines, difference maps are produced. The RGB parameters were collected in the center-half of each spot. Chemometric analysis (i.e., principal component analysis (PCA) and hierarchical cluster analysis (HCA)) is applied on the RGB triplet.

These sensor arrays demonstrated the ability to discriminate among eleven similar aliphatic amines and gaseous ammonia at different concentrations. The detection limits are always below the permissible exposure limit (50 ppm).

As(V) poisoning provokes severe damage to human health and the environment; indeed, arsenic contamination in groundwater is a significant problem in several countries. The exposure of this pollutant can cause severe health hazards such as skin discoloration and thickening, lesions, nausea, diarrhea, paralysis, blindness, and lung and bladder cancer. Arsenic toxicity largely depends on its chemical forms: Arsenite and arsenate are the most toxic species.

A low-cost, colorimetric, hydrogel, dipstick device for As(V) is developed [100]; the method consists of forming a blue-colored antimonyl–arseno–molybdate complex. The sensor is obtained by encapsulating ammonium molybdate, potassium antimonyl tartrate, and ascorbic acid in a polymer hydrogel of acrylamide, polyvinyl alcohol, and glutaraldehyde. The as-prepared hydrogel is dip-coated with plastic detector strips.

In particular, the method is based on arsenate’s reaction with ammonium molybdate, which forms an arseno–molybdate blue complex in the presence of a reducing agent (for example, L-ascorbic acid).

**Figure 17:**
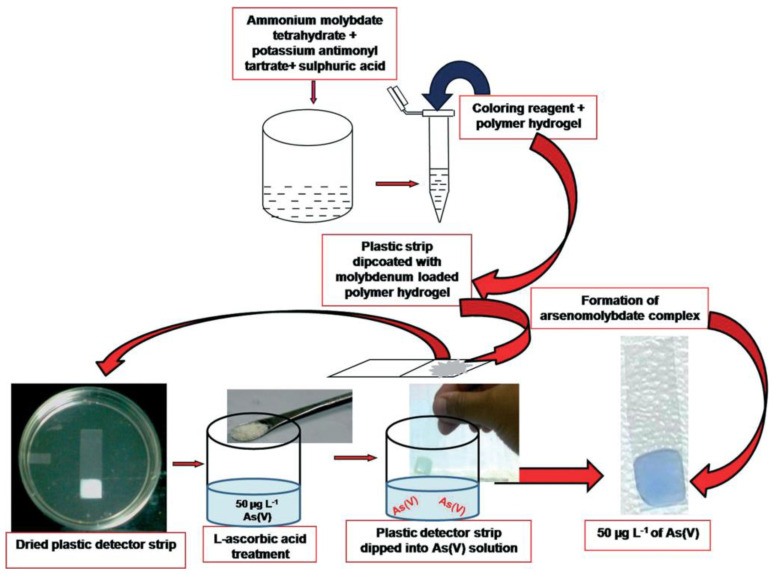
Schematic diagram of the whole procedure for As(V) determination by the polymer hydrogel-based colorimetric dipstick sensor described in [100].

Figure 17 shows a schematic of the whole process, from the synthesis to the sensing.

This device has a pretty good LOD, lower than 10 μg L^−1^ of As(V); moreover, it displayed good selectivity in the presence of <100 μg L^−1^ of phosphate and 3 mg L^−1^ of iron. This sensor is simple, portable, cheap, and user-friendly for on-site determination. The RGB analysis gave impressive results, since the As(V) concentration can be simply determined by digital analysis of the picture taken by a smartphone camera, and without any instrument, power, or other utilities.

Among all the transition metal ions, iron is an essential element in different biochemical processes, such as in oxygen transportation and cellular metabolism. On the other hand, iron deficiency or overdose results in abnormal dangerous functions in the body’s system and induces various diseases, such as Parkinson’s, Alzheimer’s cancer, and anemia. Therefore, there is a need for active sensors for monitoring iron content in biological and environmental samples.

To this end, a selective and reusable colorimetric sensor for visual detection of Fe(III) is developed [101]. The system consists of a hybrid polymeric film prepared by the sol-gel technique and functionalized with rhodamine derivative (RB-UTES).

The sensor film exhibited high selectivity and sensitivity to Fe(III) and allowed direct naked-eye detection, since the film’s color changes from white to pink. The sensor film returns to its original color after cleaning with 0.1 M ethylenediamine.

Thanks to its analytical performance, this sensor is promising for the environmental analysis of Fe(III).

## 5. Conclusions

Even if there is an extensive range of low-cost, disposable, colorimetric sensors already available commercially or prepared in research labs, thanks to the expansion of smartphones, digital communication networks, image analysis apps, and chemometric tools, the field of this kind of sensing still has much room to grow.

There is a need to develop “zero-cost” disposable sensors interfaced with open-source software and hardware.

Future trends should comprise: New devices obtained using “green” materials; low-cost, sustainable, and biodegradable sensors; miniaturization and use of portable analyzers or smartphones; and application of functionalized nanomaterials.

Moreover, smartphone-based colorimetric devices’ development aims to strengthen more handy and practical environmental analytes testing in resource-limited zones.
